# Supplementary material for: Combinatorial inhibition of LSD1 and Menin induces therapeutic differentiation in AML
Source: bioRxiv. 2025 Nov 11:2025.11.09.687496. Preprint. [Version 1] doi: 10.1101/2025.11.09.687496 (PMC12642425; doi:10.1101/2025.11.09.687496)
Supplement: 1 [file NIHPP2025.11.09.687496v1-supplement-1.pdf]

bioRxiv preprint doi: https://doi.org/10.1101/2025.11.09.687496; this version posted November 11, 2025. The copyright holder for this preprint (which was not certified by peer review) is the author/funder, who has granted bioRxiv a license to display the preprint in perpetuity. It is made available under aCC-BY 4.0 International license.

Supplemental Figure Legends

Figure S1. LSD1i synergy screen design. A. Schematic showing scheme for differentiation-specific chromatin-focused screen for genes whose inhibition synergizes with ORY-1001 to induce differentiation as gauged by CD11b as a differentiation readout.

Figure S2. LSD1 inhibition synergizes with Menin inhibition to induce differentiation. A. CD11b differentiation response assays in MLL-AF9 cells treated with GSK-LSD1, SNDX-5613, the combination, or neither. **B.** Superoxide anion response of MLL-AF9 cells upon treatment with ORY-1001, SNDX-5613, the combination, or neither.

A

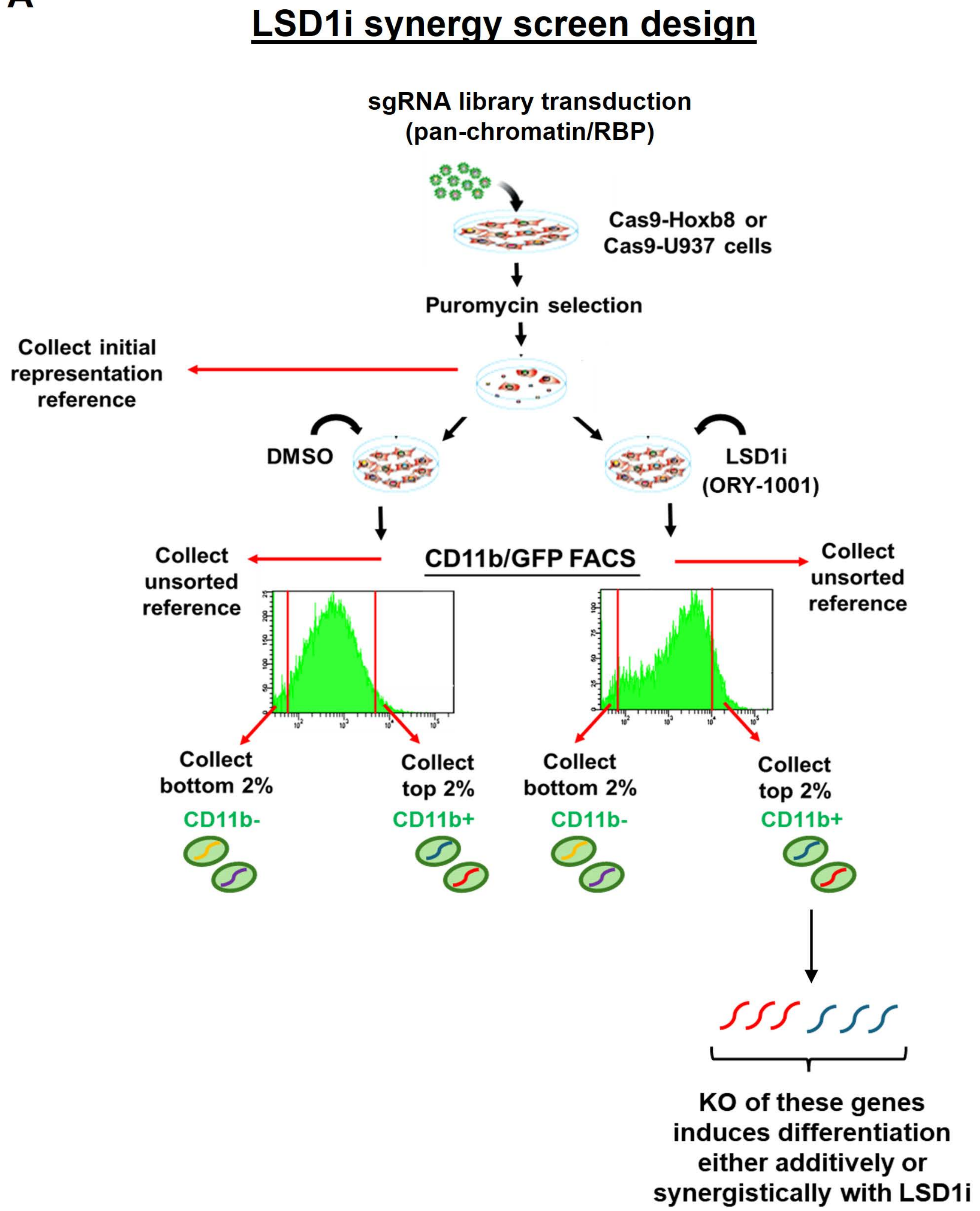

LSD1i synergy screen design
sgRNA library transduction
(pan-chromatin/RBP)
Cas9-Hoxb8 or
Cas9-U937 cells
Puromycin selection
Collect initial
representation
reference
DMSO
LSD1i
(ORY-1001)
CD11b/GFP FACS
Collect
unsorted
reference
Collect
unsorted
reference
Collect
bottom 2%
CD11b-
Collect
top 2%
CD11b+
Collect
bottom 2%
CD11b-
Collect
top 2%
CD11b+
KO of these genes
induces differentiation
either additively or
synergistically with LSD1i

Figure S1

A

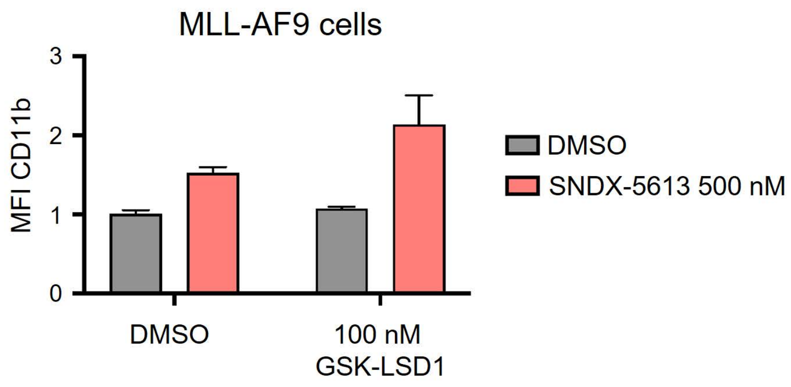
MLL-AF9 cells
MFI CD11b
3
2
1
0
DMSO
100 nM
GSK-LSD1
DMSO
SNDX-5613 500 nM

B

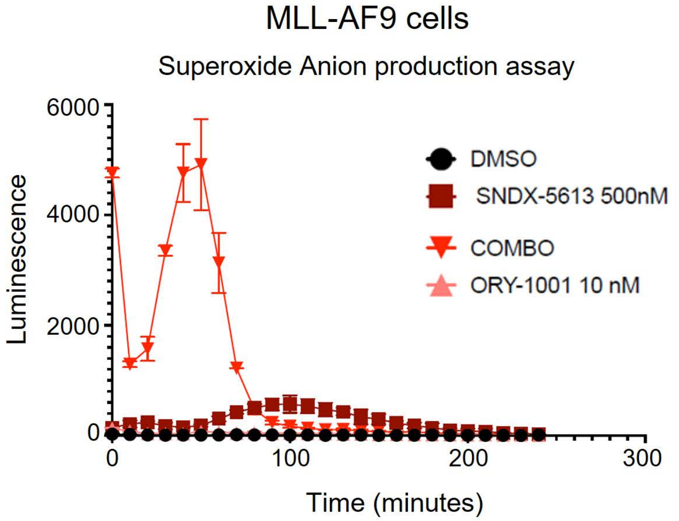
MLL-AF9 cells
Superoxide Anion production assay
Luminescence
6000
4000
2000
0
0
100
200
300
Time (minutes)
DMSO
SNDX-5613 500nM
COMBO
ORY-1001 10 nM

Figure S2
